# Supplementary material for: How do children overcome their pragmatic performance problems in the true belief task? The role of advanced pragmatics and higher-order theory of mind
Source: PLoS One. 2022 Apr 27;17(4):e0266959. doi: 10.1371/journal.pone.0266959 (PMC9045612; doi:10.1371/journal.pone.0266959)
Supplement: S1 File — (PDF) [file pone.0266959.s001.pdf]

Supplementary Material

Study 1

Syntactic Recursion Task (adapted from Arslan et al., 2017)

Figure 1

Trial 1 of the Syntactic Recursion Task, 1. order recursion

- Practice: Where is the horse?

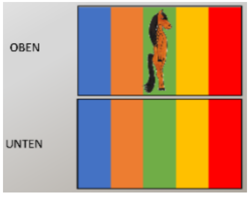

- Practice: Where is the horse that strokes?

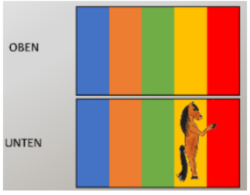

- Practice: Where is the cow?

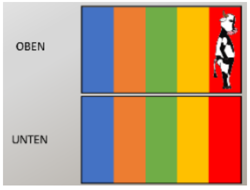

- Practice: Where is the cow that strokes?

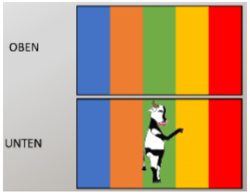

- Control question: Where is the horse that strokes?

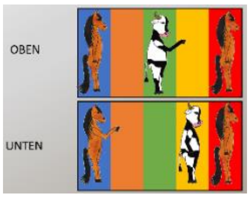

- Test question: Where is the cow that strokes a horse?

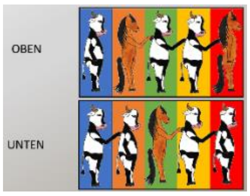

Trial 2: 2. order recursion

- Practice: Where is the dog?
- Practice: Where is the dog that nudges?
- Practice: Where is the cat?
- Practice: Where is the cat that nudges?
- Control question: Where is the dog that nudges?
- Test question: Where is the cat that nudges a dog that nudges a cat?

Trial 3: 3. order recursion*

- Practice: Where is the monkey?
- Practice: Where is the monkey that bites?
- Practice: Where is the lion?
- Practice: Where is the lion that bites?
- Control question: Where is the monkey that bites?
- Test question: Where is the lion that bites a monkey that bites a lion that bites a lion?

Trial 4: 4. order recursion*

- Practice: Where is the bunny?
- Practice: Where is the bunny that tickles?
- Practice: Where is the mouse?
- Practice: Where is the mouse that tickles?
- Control question: Where is the bunny that tickles?
- Test question: Where is the mouse that tickles the bunny that tickels the mouse that tickles a bunny that tickles a mouse?

**For the 3. and 4. order recursion trial, the row of animals was complemented with a sixth color (purple) to have more animals on the picture than in the sentence.*

Note: Practice questions were included for two reasons. First, practice questions trained the child to answer this kind of questions with the combination of the background color and the row. Second, children learnt to differentiate between an anima that does not perform an action and an animal that performs an action (e.g. tickles, nudges, etc.). In the very first trial, E helped the child to answer the practice questions with a combination of both if necessary and corrected all practice questions if wrong. For the practice question, only one animal was depicted on the screen. The picture for the control question always contained five to six animals so that children had to differenciate the two animal species and the action learnt during the practice. The picture for the test question depicted an animal on each background color (10 animals for trial 1 and 2, 12 animals for trial 3 and 4).

Standard change-of-location Task (adapted from (Rakoczy & Oktay-Gür, 2020)Wimmer & Perner, 1983)

Figure 2

True belief trial 1

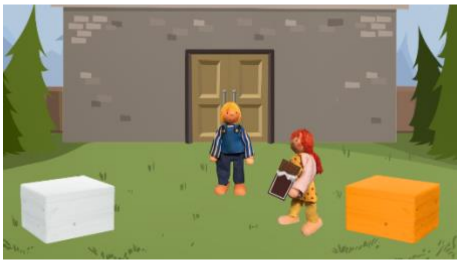

This is Leon with some chocolate. Leon has to go and wash his hands. Therefore, he puts the chocolate into the yellow box. Then, Leon goes into the house. Leon is in the house to wash his hands. Then, Leon comes back. And Caro comes, too. Caro takes the chocolate out of the yellow box and puts it into the white box. Leon is watching Caro doing that. Then, Caro leaves.

- Test question: What does Leon think where the chocolate is?
- Control question 1: In which box was the chocolate first?
- Control question 2: In which box is the chocolate now?

False belief trial 1

This is Eva with her toy car. Eva is bored, so she wants to go inside to play something. She puts her car into the yellow box. Then, Eva goes into the house. Eva is in the house to play something inside. Then, Felix comes. Felix takes the car out of the purple box and puts it into the green box. Then, Felix leaves. Now, Eva comes back.

- Test question: What does Eva think where the toy car is?
- Control question 1: In which box was the car first?
- Control question 2: In which box is the car now?

True belief trial 2

This is Nina with her teddy bear. Nina has to go to the toilet. Therefore, she puts her teddy bear into the yellow box. Then, Nina goes into the house. Nina is in the house to go to the toilet. Then, Nina comes back. And Till comes, too. Till takes the teddy bear out of the yellow box and puts it into the pink box. Nina is watching Till doing that. Then, Till leaves.

- Test question: What does Nina think where the teddy bear is?
- Control question 1: In which box was the teddy bear first?
- Control question 2: In which box is the teddy bear now?

False belief trial 2

This is Tine with her ball. Eva is thirsty, so she wants to go and drink something. She puts her ball into the blue box. Then, Tine goes into the house. Tine is in the house to drink something. Then, Mark comes. Mark takes the ball out of the blue box and puts it into the red box. Then, Mark leaves. Now, Tine comes back and wants to play with her ball.

- Test question: What does Tine think where the ball is?
- Control question 1: In which box was the ball first?
- Control question 2: In which box is the ball now?

<u>Task for Recursive Theory of Mind Understanding</u> (adapted from Liddle & Nettle, 2006)

Story 1: The school football team

This is Max and this is Paul. “Hi, Im Paul”, “and im Max!“. Max and Paul are best friends. They really enjoy playing football together. Max and Paul both want to play on the school footbal team. The school football team plays every Monday after school. Max thinks that he is not as good at football as Paul is. He thinks that the football manager only chooses Paul for the football team. But the football manager thinks that both Max and Paul are good football players. He wants them both to play in the school football team. But the manager knows that Max doesn’t think he will get on the team.

Memory question

Which sentence is correct?

a) The football team play on Mondays after school.

b) The football team play on Fridays after school.

ToM Level 2

Which sentence is correct?

a) Max doesn’t know that the manager wants that both him and Paul play on the team.*

b) Max knows that the manager wants that both him and Paul be on the team.*

Memory question

Which sentence is correct?

a) Max and Paul are brothers.

b) Max and Paul are best friends.

ToM Level 3

Which sentence is correct?

a) The manager thinks that Max believes that he (the manager) wants that Max is on the team.

b) The manager thinks that Max believes that he (the manager) wants that Max is not on the team.

Story 2: The video dilemma

This is Sarah and this is Olli. Sarah and Olli are in the same class at school. “Hi, I’m Sarah!’, “and I’m Olli”. Their teacher is Mrs. Brown. Today Mrs. Brown suggests that Sarah and Olli should bring a video in to school tomorrow to watch with the other children. Mrs. Brown also says to them, “Make sure you bring a film that I will like too!” (Mrs. Brown leaves the scene). Sarah’s favorite videos are pirate videos. Olli’s favorite videos are horse films. Which will it be? A pirates or a horse film? Olli says to Sarah, “We just can’t decide so I think that we should take in the film that Mrs. Brown would like. Sarah, do you know which Mrs. Brown would like best?” Sarah has a little think. She does not have a clue which film Mrs. Brown would like! But Sarah decides to tell Olli that she knows that Mrs. Brown likes pirate films best. Sarah thinks that this will make Olli agree to take a pirate video in to school. Olli listens to this and then Olli says; “We will take in a video of pirates then.” So, Sarah gets to enjoy her favorite film!

Memory question

Which sentence is correct?

a) Sarah likes pirates films best.

b) Sarah likes horse films best.

Test question (ToM Level 4)
Which sentence is correct?
a) Sarah hopes that Olli believes that she doesn’t know that Mrs. Brown wants that they watch a pirate film*.
b) Sarah hopes that Olli believes that she knows that Mrs. Brown wants that they watch a pirate film.*

Story 3: The birthday cake

Ben, Paula and Otto are in the same class at school. “Hello, I’m Ben”, “and I am Paula”, “I’m Otto, hello!”. Ben likes Paula a lot. He knows that it is Paula’s birthday tomorrow and that she loves strawberry cake. Ben wants to bake a strawberry cake for her birthday. The next day, Ben brings the strawberry cake to school. Paula is not in the classroom, yet. Only Otto is there. Otto’s desk is next to Paula’s in the classroom. Ben puts the cake secretly as a surprise on Paula’s desk and says to Otto “that’s my birthday surprise for Paula”. Then, Ben runs quickly out of the classroom. Otto likes Paula, too. But he forgot about her birthday. He has no present for her. Otto thinks that Ben is not around. He writes on a piece of paper “from Otto” and puts the piece of paper next to the cake. But Ben was secretly behind the shelf and saw everything Otto did.

Memory question
Which sentence is correct?

- Correct: Ben baked the cake for Paula.
- Incorrect: Otto baked the cake for Paula.

Test question (ToM Level 5)
Which sentence is correct?

- Incorrect: Ben knows that Otto thinks that Ben knows that Otto wants that Paula believes that the cake is from Otto.
- Correct: Ben knows that Otto thinks that Ben does not know that Otto wants that Paula believes that the cake is from Otto.

*Direct translation from German language with that-complement for the verb “want” (“möchte, dass”)

Advanced Pragmatics Task

The playing girl

- Picture 1: Lisa is running through the apartment all day. She plays with the ball, jumps on the sofa and plays tag with the cat.
- Metaphor question: What fits the best? Lisa is…
    - A cloud
    - A crocodile
    - A whirlwind* (*Correct Answer: Metaphor used in German for very active children)
    - A tree
- Picture 2: In this moment, Lisa is running so fast that she hits the table and all the books fall on the ground.
- Picture 3: Lisa’s older brother enters the rooms and says, “You’re very careful today.”
    - Irony test question 1: Does the brother want Lisa to believe that he thinks that she was careful?
    - Irony test question 2: Why does the brother say, “You’re very careful today again”?
    - Control question: Does the brother find that Lisa was careful?

Figure 3

3 Pictures used for the story “the playing girl” in the Pragmatic Language Task. Pictures were presented separately. The child saw picture 1 during the metaphor test question. Picture 3 was depicted during the irony questions.

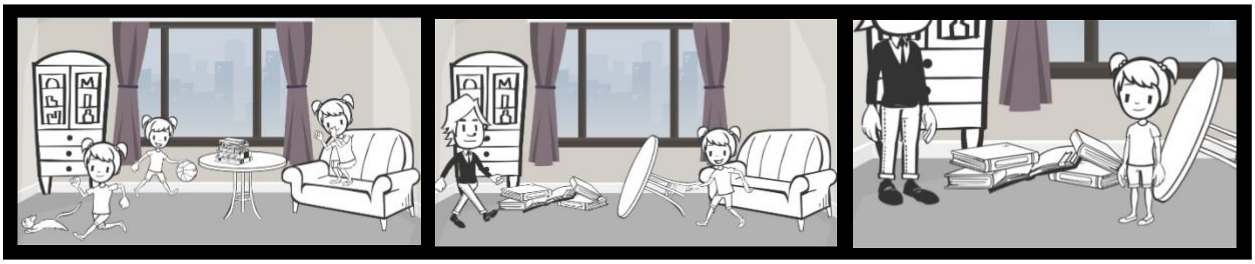

The walk in the snow

- Picture 1: Simon goes for an hour-long walk in the snow. He is just about coming back home.
- Metaphor question: What fits the best? Now, Simon’s feet are…
    - Hooves
    - Icicles* (*Correct Answer: Metaphor used in German for cold parts of the body)
    - Cheese
    - Stones
- Picture 2: Simon's mother comes out of the house and sees that Simon was walking in the snow in slippers.
- Picture 3: Simon's mother says to Simon, “That was very clever of you.”
    - Irony test question 1: Does the mother want Simon to believe that she thinks that that was clever of him?
    - Irony test question 2: Why does the mother say, “That was very clever of you”?
    - Control question: Does the mother think that this was clever of him?

Additional information for pragmatic language task

After presenting the first picture, the metaphor test question was asked (“What fits the best?”). The children had to find the correct answer out of four answer options respective to the story line. The answer options could be repeated up to 4 times. The correct answer option contained a suitable metaphor (e.g. “icicles” to figuratively describe the agent’s cold feet after a long walk in the snow). The three distracting answer option contained related words that are not typically used as a metaphor in this context (e.g. hooves). After the metaphor test question, the story continued with the remaining two pictures. After the third picture, the story ended with an ironic utterance of the older agent (mother, older brother) criticizing the other one (son, little sister). The speaker’s face was not visible on the third picture to avoid any inferences from their facial expression. Following this, two irony test questions were posed. The first test question referred to the speaker’s intention in uttering an ironic comment. (“Does *the speaker* want the listener to believe that he (*the speaker*) thinks that [*literal meaning of the utterance*]?”). The second question targeted the children’s understanding of the irony concept. (“Why did the *speaker* say [*ironic utterance*]?”). To answer the second test question correctly, children had to refer to the speaker’s mental state or attitude to the other agent’s behavior or refer to the negative outcome of the other agent’s behavior or to the opposite/ ironic meaning of the utterance. Answers to

this test questions were coded with a fixed coding scheme (partly adapted from Angeleri & Airenti, 2014; Filippova & Astington, 2008). The two irony test questions were followed by a control question (“Does the speaker think that [literal meaning of the ironic utterance]?”).

Table 1

Coding Scheme for Irony Test question 2 (partly adapted from Angeleri & Airenti, 2014; Filippova & Astington, 2008)

Category	Subcategory	Theoretic Definition	Operational Definition	Repetition of test question
**Incorrect (0)**	A No understanding	No explanation	„I don’t know“	Yes
		Inappropriate explanation	„She made a mistake“ „She said it accidentally“	No
		Explanation with irrelevant information	„This is her brother“	Yes
	B Literal understanding	Literal interpretation	„Because she thinks that Simon was clever“	No
		Repetition of utterance	„That was very clever of you, Simon“	Yes
	C Conventions	Learnt, conventionalized explanations	„Because you don’t do that“ „Because he is not supposed to do that“	Yes
	D Insufficient explanation	Repetition of the corresponding metaphor	„She is a whirlwind“	Yes
**Correct (1)**	A Mental state	Reference to speaker’s second-order intention	„So that he thinks that she does not think that…“	No
		Reference to mental state	„Because she thinks that was not clever at all“	No
	B Attitude	Reference to speaker’s attitude (critism, mocking, joking)	„Because she does not like him to do that“ „He was joking“	No
	C Outcome	Reference to negative outcome of the action	„So that he does not get ill“ „So that he won’t do it again“	No
	D Denotation of Irony	Reference to irony	„That was ironic“ „She meant the exactly opposite meaning“	No

Table 2

Detailed summary of answers to irony test question 2

		Snow Story			Girl Story		
**Category**	**Sub-category**	*n*	*n* noTB	*n* TB	*n*	*n* noTB	*n* TB
**Incorrect (0)**	A No understanding	20	6	14	19	5	14
	B Literal understanding	2	1	1	0	0	0
	C Conventions	1	1	0	1	1	0
	D Insufficient explanatation	0	0	0	1	1	0
**Correct (1)**	A Mental state	13	6	7	8	3	5
	B Attitude	4	4	0	7	7	0
	C Outcome	5	4	1	15	13	2
	D Denotation of Irony	33	10	23	28	3	25

Study 2

Recusive ToM Production task

Story 1: The ice-cream van

This is Bob, and this is Susi, they are brother and sister. As Bob comes home from school today, he tells Susi that he saw the ice-cream van on his way back. Both Susi and Bob like ice-cream very much and they want to buy some ice-cream from the ice-cream van this afternoon. The ice-cream van is always either in the park or by the lake.
But Susi and Bob have to do their homework first. Susi finishes her homework earlier than Bob. Bob doesn't want Susi to get the ice-cream earlier than he does. Bob knows that the ice-cream van is by the lake today because he saw it there on his way back from school. Susi asks Bob where the ice-cream van is today. Bob answers "the ice cream truck is at the park today."

Control question: Is that right what he said?
Test Question: Why did he say that?
For follow-up test question depending on length of the answer to initial test question:

- Child does not answer/ answers "I don't know": "Think again. Why does he say that? You certainly have a good idea."
- Child gives short answer (up to about 15 words): "That's a good idea. Can you explain this to me in more detail?"
- Child gives detailed answer (more than 15 words): "That's a good idea. Do you want to add something or should we go on with the next story?"

Story 2: The treasure diggers

In the battle between the pirates and the treasure diggers, the pirates hold one of the treasure diggers captive. The pirates want the captured treasure digger to tell them where the treasure of the treasure diggers is. The camp with the treasure is either on the island or in the mountains. They know that the captive knows where the camp is, but he will not want to tell them. He wants to save his army, so he will surely lie to the pirates.

The prisoner is very brave and very smart, he will not let them find the treasure. The camp with the treasure is in the mountains. Now, when the pirates ask where the camp with the treasure is, the prisoner answers, "In the mountains."

Control question: Is that right what he said?
Test Question: Why did he say that?
Follow-up question (*see* above)

Story 2 continued

The pirates were very busy searching for the treasure. Thus, the treasure digger successfully freed himself from the captivity of the pirates and returned to his fortress. The pirates still have not found the treasure. They now know that the treasure must really have been in the mountains when the treasure digger told them.
Two weeks later, the fortress is again attacked by the pirates and the same treasure digger is again captured by the pirates. In the meantime, the treasure diggers have re-hidden the treasure. The pirates still want the treasure of the treasure diggers. They ask the captive again where the treasure is. The pirates know that this time the treasure is either in the field or at the harbor. The prisoner, of course, still doesn't want the pirates to find the treasure. The pirates are not very smart, they expect the treasure digger to answer the same way as last time. The camp with the treasure is in the field. When the pirates ask where the camp with the treasure is, the prisoner answers "at the harbor".

Control question: Is that right what he said?
Test Question: Why did he say that?
Follow-up question (*see* above)

Recursive Theory of Mind Understanding

New story lines and questions for the task for (partly adapted from Henzi et al., 2007; Liddle & Nettle, 2006)

Story 2: The video dilemma

Memory question:

Which sentence is correct?

- Incorrect: Sarah and Olli go to different school classes.
- Correct: Sarah and Olli go to the same school class.

Which sentence is correct? (ToM Level 2)

- Incorrect: Sarah knows that Olli wants them to watch a pirate film.
- Correct: Sarah knows that Olli wants them to watch a horse film.

Story 3: The birthday cake

Memory question:

Which sentence is correct?

- Incorrect: Paula does not like strawberry cake.
- Correct: Paula likes strawberry cake.

Test question (TOM Level 4)

Which sentence is correct?

- Correct: Otto hopes that Paula thinks that he knows that Paula wants her to have a strawberry cake for her birthday.
- Incorrect: Otto hopes that Paula thinks that he doesn't know that Paula wants her to have a strawberry cake for her birthday.

New Story line 4: The eggs accident

These are the siblings Tobi and Lucy with their mother. Today the mother comes home with a big shopping. She has bought a lot of eggs because they are going to have fried eggs for dinner. The mother puts the basket with the eggs on the kitchen shelf and goes out into the garden. While the mother is outside, Tobi comes into the kitchen. Tobi is curious and looks in

the basket and accidentally knocks over the basket with the eggs. All the eggs are on the floor and broken. Tobi thinks about not telling anyone what happened to him and starts to clean up the eggs. At that moment, the mother comes back into the kitchen and sees Tobi wiping up the eggs. Tobi tells the mother that Lucy broke the eggs and he is just cleaning up the eggs for her. But the mother knows that Tobi is lying because Lucy was in the garden with her the whole time.

Memory question

Which sentence is correct?

- Incorrect: Tobi and Lucy are neighbors.
- Correct: Tobi and Lucy are siblings.

Test question (ToM Level 3)

Which sentence is correct?

- Correct: Tobi thinks that his mother believes him that he knows that Lucy broke the eggs.
- Incorrect: Tobi thinks that the mother doesn't believe him that he knows that Lucy broke the eggs.
-

Memory question

Which sentence is correct?

- Incorrect: Lucy did not break the eggs.
- Correct: Lucy break the eggs.

Test question (ToM Level 5)

Which sentence is correct?

- Correct: The mother knows that Tobi doesn't know that she knows that Tobi hopes that the mother believes that Lucy broke the eggs.
- Incorrect: The mother knows that Tobi knows that she knows that Tom hopes that the mother believes that Lucy broke the eggs.

Interrater Reliabilities

	Task	Cohen's κ
Study 1	Syntactic Recursion	.92
	TB trial 1	1
	TB trial 2	1
	Recursive ToM Understanding	.91
	Metaphor	1
	Irony question 1	1
	Irony question 2	.83
Study 2	Syntactic Recursion	.94
	TB trial 1	1
	Recursive ToM Production	.91
	Recursive ToM Understanding	.94
Study 3	Syntactic Recursion	.91
	TB trial 1	1
	Recursive ToM Understanding	1

References

Angeleri, R., & Airenti, G. (2014). The development of joke and irony understanding: A study with 3- to 6-year-old children. *Canadian Journal of Experimental Psychology*, *68*(2), 133–146. https://doi.org/10.1037/cep0000011

Arslan, B., Hohenberger, A., & Verbrugge, R. (2017). Syntactic recursion facilitates and working memory predicts recursive theory of mind. *PLoS ONE*, *12*(1), 1–23. https://doi.org/10.1371/journal.pone.0169510

Filippova, E., & Astington, J. W. (2008). Further development in social reasoning revealed in discourse irony understanding. *Child Development*, *79*(1), 126–138. https://doi.org/10.1111/j.1467-8624.2007.01115.x

Henzi, S. P., de Sousa Pereira, L. F., Hawker-Bond, D., Stiller, J., Dunbar, R. I. M., & Barrett, L. (2007). Look who’s talking: Developmental trends in the size of conversational cliques. *Evolution and Human Behavior*, *28*(1), 66–74. https://doi.org/10.1016/j.evolhumbehav.2006.07.002

Liddle, B., & Nettle, D. (2006). Higher-Order Theory of Mind and Social Competence in school-age Children. *Journal of Cultural and Evolutionary Psychology*, *4*, 215–229. https://doi.org/10.1556/JCEP.4.2006.3

Wimmer, H., & Perner, J. (1983). Beliefs about beliefs: Representation and constraining function of wrong beliefs in young children’s understanding of deception. *Cognition*, *13*(1), 103–128. https://doi.org/10.1016/0010-0277(83)90004-5
