# Supplementary material for: How do children overcome their pragmatic performance problems in the true belief task? The role of advanced pragmatics and higher-order theory of mind
Source: PLoS One. 2022 Apr 27;17(4):e0266959. doi: 10.1371/journal.pone.0266959 (PMC9045612; doi:10.1371/journal.pone.0266959)
Supplement: Attachment — Submitted filename: PLOS ONE_review.pdf [file pone.0266959.s005.pdf]

Review: How do children overcome their pragmatic performance problems in the True Belief Task? The Role of Advanced Pragmatics and Higher-order Theory of Mind

The manuscript presents three experimental studies with 6- to 10-year-olds. The aim of these experiments is to assess whether children's performance in a standard True-belief (TB) task can be predicted by their pragmatic skills, their recursive ToM skills, or more general recursive abilities.

This investigation addresses an open question in developmental research: why do children's performance in the TB task is characterized by a U-shaped developmental trajectory? More specifically, it focuses on the question of which cognitive skills determine the end of the U-shaped performance curve.

The studies are methodologically rigorous and well described, the statistical analyses are appropriately reported, and they certainly deserve to be published in PLOS ONE.

I have some recommendations to revise the manuscript before publication, which I list below. These concern the Introduction and the General discussion, and more specifically, the presentation and discussion of the notion of 'advanced pragmatic abilities', and their alleged role in the experimental puzzle under discussion.

First of all, I believe the introduction should include a more explicit description of the pragmatic analysis of children's performance in the TB task, and the extent to which the analysis provided meshes well with the available experimental evidence in developmental pragmatics.

The authors suggest that "once children develop the prerequisite ToM capacities, they develop an understanding for pragmatics" (149-150) Before that, they would "mostly use and interpret language literally" (139). This analysis offers an overly-simplified picture of pragmatic development. On the one hand, recent work in pragmatics shows that children can interpret language non-literally before the age at which they typically pass the standard FB task. For instance, work from Nausicaa Pouscoulous and colleagues has shown that children are able to understand metaphors already at the age of 3 (see, e.g., Pouscoulous & Tomasello, 2020). Similar results have been found when looking at metonymy understanding, which also displays an interesting U-shaped curve (see, e.g., Falkum, Recasens & Clark, 2017; Köder & Falkum, 2020). On the other hand, some pragmatic phenomena, such as irony understanding, emerge later in development (for a discussion, see Pexman, f*orthcoming*).

Given the relevance of pragmatic skills to the issue under discussion, I suggest reviewing in more detail the experimental literature on the development of pragmatics. Most importantly, the authors should give more prominence to the distinct developmental trajectories that characterize different pragmatic phenomena, without assimilating metaphor and irony. This issue is particularly salient in the section "Advanced pragmatics understanding" where these phenomena are discussed under the umbrella term "indirect speech acts", which would "involve a mismatch of sentence and speaker meaning" (208-209).

Furthermore, this section includes a very brief mention of the literature in clinical pragmatics and suggests that the pragmatic difficulties shown by individuals with ASD are fundamentally related to their ToM impairments. As this issue is vigorously debated in the current pragmatic literature, I suggest a more nuanced and informed discussion of the relevance of data from ASD

to the issue under discussion (see, e.g., Andrés-Roqueta & Katsos, 2017; Kissine 2021; Mazzarella & Noveck, 2021).

Finally, I think the authors should more convincingly discuss *what kind* of advanced pragmatic abilities are expected to be involved in the TB task and *why*, and, in light of this, better justify the choice of the selected measurements (metaphor and irony understanding).

These changes in the Introduction should also be reflected in a revision of the General discussion.

Minor comments:

126-129: It would be useful to include more references to empirical data showing that "academic question formats are difficult to grasp for young children".

Fig. 2 The visual animation seems very difficult to interpret, and it does not strike as an intuitive representation of the story "the video dilemma". Could you please better justify the choice of this visual representation format and spell out more explicitly its appropriateness for the target age groups?

Typos:

354: Delete "were"

717: Delete "that"

References

Andrés-Roqueta, C., & Katsos, N. (2017). The contribution of grammar, vocabulary and theory of mind in pragmatic language competence in children with autistic spectrum disorders. *Frontiers in psychology*, *8*, 996.

Falkum, I. L., Recasens, M., & Clark, E. V. (2017). "The moustache sits down first": on the acquisition of metonymy. *Journal of Child Language*, *44*(1), 87-119.

Kissine, M. (2021). Autism, constructionism and nativism. *Language*, 97(3), e139-e160.

Köder, F., & Falkum, I. L. (2020). Children's metonymy comprehension: Evidence from eye-tracking and picture selection. *Journal of Pragmatics*, *156*, 191-205.

Mazzarella, D., & Noveck, I. (2021). Pragmatics and mind reading: The puzzle of autism (Response to Kissine). *Language*, *97*(3), e198-e210.

Pexman, P. M. (forthcoming) Irony and Thought: Developmental Insights. *Handbook of irony and thought.* Available here: https://www.researchgate.net/profile/Penny-Pexman/publication/350740252_Irony_and_Thought_Developmental_Insights/links/606f5214299bf1c911ba1101/Irony-and-Thought-Developmental-Insights.pdf

Pouscoulous, N., & Tomasello, M. (2020). Early birds: Metaphor understanding in 3-year-olds. *Journal of Pragmatics*, *156*, 160-167.
